# Transplacental transmission of Human Papillomavirus

**DOI:** 10.1186/1743-422X-5-106

**Published:** 2008-09-25

**Authors:** Renato L Rombaldi, Eduardo P Serafini, Jovana Mandelli, Edineia Zimmermann, Kamille P Losquiavo

**Affiliations:** 1 Diagnosis – Molecular Laboratory, University of Caxias do Sul, Caxias do Sul, Rio Grande do Sul, Brazil; 2 Pathology Medical Laboratory, Department of Health and Biomedical Science, University of Caxias do Sul, Caxias do Sul, Rio Grande do Sul, Brazil; 3 Biotechnology Institute, University of Caxias do Sul, Caxias do Sul, Rio Grande do Sul, Brazil; 4 Outpatient Clinic of Genital Pathology, Department of Clinical Medicine, University of Caxias do Sul, Caxias do Sul, Rio Grande do Sul, Brazil

## Abstract

This paper aimed at studying the transplacental transmission of HPV and looking at the epidemiological factors involved in maternal viral infection. The following sampling methods were used: (1) in the pregnant woman, (a) genital; (b) peripheral blood; (2) in the newborn, (a) oral cavity, axillary and inguinal regions; (b) nasopharyngeal aspirate, and (c) cord blood; (3) in the placenta. The HPV DNA was identified using two methods: multiplex PCR of human β-globin and of HPV using the PGMY09 and PGMY11 primers; and nested-PCR, which combines degenerated primers of the E6/E7 regions of the HPV virus, that allowed the identification of genotypes 6/11, 16, 18, 31, 33, 42, 52 and 58. Transplacental transmission was considered when type-specific HPV concordance was found between the mother, the placenta and the newborn or the mother and cord blood. The study included 49 HPV DNA-positive pregnant women at delivery. Twelve placentas (24.5%, n = 12/49) had a positive result for HPV DNA. Eleven newborn were HPV DNA positive in samples from the nasopharyngeal or buccal and body or cord blood. In 5 cases (10.2%, n = 5/49) there was HPV type-specific agreement between genital/placenta/newborn samples. In one case (2%, n = 1/49) there was type specific HPV concordance between genital/cord blood and also suggested transplacental transmission. A positive and significant correlation was observed between transplacental transmission of HPV infection and the maternal variables of immunodepression history (HIV, p = 0.011). In conclusion the study suggests placental infection in 23.3% of the cases studied and transplacental transmission in 12.2%. It is suggested that in future HPV DNA be researched in the normal endometrium of women of reproductive age. The possible consequence of fetal exposure to HPV should be observed.

## Background

Human papillomavirus (HPV), the most common sexually transmitted infection, has been recognized as a cause of anogenital warts (HPV type 6 and 11) and cervical cancer (HPV type 16, 18 and others)[1]. In children, HPV-related (type 6 and 11) laryngeal papillomas, conjunctival papillomas and genital warts [2-6].

Although it has been established that HPV is sexually transmitted[7,8], there is growing evidence that non-sexual transmission also occurs[9]. This includes vertical transmission from parents to infants, horizontal transmission from other family members and those in close contact with the child, autoinoculation from one site to another and possibly indirect transmission via phomites[10]. The potential mother-to-child HPV transmission route in the perinatal period has been demonstrated [11-17]. There is evidence of vertical transmission, presumably occurring during passage of the fetus through an infected birth canal[18]. The virus could also be transmitted by ascending infection, especially after premature rupture of the membranes. In utero transmission could be caused either by ascending infection from an infected birth canal, by sperm at fertilization or hematogenously (transplacentally). HPV DNA has been detected in peripheral blood mononuclear cells of pregnant women[19], cord blood specimens of neonates[19], oropharyngeal secretions of neonates[20], amniotic fluid [21-23], fetal membranes[24], placental trophoblastic cells[11], infants born by elective cesarean section delivery[11,13,18,22,24], and in syncytiotrophoblastic cells of spontaneously aborted material[25]. In addition, there are type-discordant cases between mothers and newborns, suggesting that many of these infants did not acquire the HPV from their mothers[26]. These observations could explain the transplacental transmission of HPV from an infected mother to the fetus. However, only a limited number of women have been studied to confirm placental transmission of HPV.

This cross-sectional, prospective study aimed at evaluating transplacental transmission of HPV and enhancing understanding of the maternal epidemiologic features involved.

## Methods

### Population studied

Between April 2005 and April 2007, a cross-sectional, prospective study was performed on 71 pregnant women (mean age 24.6 ± 7.7 years, 14–41 years) with a prior history of HPV infection (n = 22), or who had abnormal Papanicolaou smear (n = 20) or genital warts (n = 29), due to the high probability that they could have HPV infection. The women were referred from the Obstetrical Service of the University of Caxias do Sul and by the Basic Health Units of the Single Health System in Caxias do Sul. This study was performed with the approval of the Ethics in Research Committee at the University of Caxias do Sul, and of the Editorial and Scientific Board of the General Hospital of Caxias do Sul, and did not present a conflict of interest. The Letter of Free and Informed Consent and the epidemiological evaluation tool were obtained from all the women by individual interviews during the obstetrical examinations. Sixty-three (79.7%) of the 71 pregnant women selected who entered the study underwent delivery and 16 (20.3%) dropped out of the study.

### Epidemiological evaluation

The epidemiological study was performed taking the following variables into account: age, race, level of education, smoking, marital status, age at first sexual   intercourse, parity, number of sexual partners in lifetime, number of sexual  partners in past year, frequency of condom use with sexual partners in lifetime, frequency of condom use with sexual partners in past year, marital stability in years, history of immunodepression (HIV – acquired immunodeficiency syndrome), type of HPV lesion (genital warts, LGSIL – low-grade squamous intraepithelial lesions, HGSIL – high-grade squamous intraepithelial lesions), site of HPV lesion (cervical, vaginal, vulvar and perineal), type of HPV infection (single, double and multiple), gestational age at the time HPV infection was diagnosed (weeks), duration of labor (minutes), time of amniotic membrane rupture (minutes), type of delivery (cesarean section, vaginal and vaginal with forceps) and HPV lesion at delivery (genital warts, LGSIL – low-grade squamous intraepithelial lesions, HGSIL – high-grade squamous intraepithelial lesions).

### Sampling methods

#### Maternal genital

The maternal genital samples were obtained during pregnancy, at the first visit, when the woman was recruited. The sample was obtained using a special brush for cytopathological sampling of the cervix, which was used for genital brushing in the following order: cervix and possible clinical and subclinical lesions of the vagina, vulva and perineal region. The brush was placed in a TE solution (Tris HCl, pH 7.5 – 10 mM; EDTA, 1 mM), and the material collected was kept frozen at -20°C, until the desoxyribonucleic acid (DNA) was extracted.

#### Peripheral blood maternal

Immediately before delivery (pre-partum period), a sample of peripheral blood was obtained from the woman using a 3 ml disposable syringe (27/5 needle), retrieving about 1 ml of blood which was placed in a KMA type tube with EDTA. The blood collected was kept frozen at -20°C, until DNA was extracted.

In newborns, in the first minutes of the life, buccal, body, nasopharyngeal aspirates and arterial blood from the umbilical cord samples were obtained.

#### Buccal and body

The swabs were collected in the first minutes of life, using the special brush for cytopathological sampling of the cervix, with which brushing was performed in the following order: buccal cavities, axillary and inguinal regions of the newborn. The brush was placed in a TE solution (Tris HCl, pH 7.5 – 10 mM; EDTA, 1 mM) and kept frozen at -20°C, until DNA was extracted.

#### Nasopharyngeal aspirates

The distal extremity of the tracheal aspiration catheter (n° 6 or 8, Sondas Descartáveis Mercosul ^® ^Linha Sondas Descartáveis Mercosul^®^, Empresa CPL Medical's Produtos Médicos LTDA), used to aspirate the upper airways (nasopharyngeal) of the newborn immediately after birth, was removed. The distal extremity of the catheter (about 4 cm long) was cut and placed in TE solution (Tris HCl, pH 7.5 – 10 mM; EDTA, 1 mM), keeping it frozen at -20°C, until DNA was extracted.

#### Arterial blood from the umbilical cord

The sample was collected directly from one of the arteries of the cord using a 3 ml disposable syringe (27/5 needle) to obtain about 1 ml of fetal blood. The collection was performed after clamping the cord and complete delivery of the placenta and fetal membranes. The fetal blood was placed in a KMA type tube with EDTA and frozen at -20°C, until DNA was extracted.

The placental sampling methods were performed immediately after complete delivery and cleaning of the placental disk sides, using surgical compresses.

#### Placental swabs

The swabs were obtained using special brushes for the cytopathological collection from the cervix, by brushing in the following order: initially on the fetal side of the placenta, and later with a new brush, on the maternal side of the placenta. The brushes were placed individually in a TE solution (Tris HCl, pH 7.5 – 10 mM; EDTA, 1 mM), keeping them frozen at -20°C, until DNA was extracted.

#### Placental biopsy

Two biopsies were performed on the sides of the placental disk: one in the more central portion; another in the more peripheral portion (placental border). The biopsies were performed with the help of the rat-tooth forceps, and the curved iris scissors. The fragments collected were placed individually in a TE solution (Tris HCl, pH 7.5 – 10 mM; EDTA, 1 mM) and kept frozen at -20°C, until DNA was extracted.

#### DNA extraction

DNA was extracted from the blood and tissue samples using the *Wizard Genomic DNA Purification Kit *(Promega), according to the manufacturer's specifications. In the brush samples, DNA was extracted using 600 μl of NaOH 50 mM stirred in a vortex for 5–10 seconds and later incubated at 95°C for 5 minutes. The solution was then neutralized with 60 μl of Tris HCl pH 8.0 and kept in a freezer at -20°C, until it was submitted to the next stages.

After the DNA extraction methodology, the products were submitted to two different PCR methods to identify and type the HPV DNA: multiplex PCR and type specific nested multiplex-PCR.

#### β-globin and HPV amplification

The DNA samples obtained using the extraction methodology were amplified in multiplex PCR, and this was composed by the PCO4 oligonucleotides (CAA CTT CAT CCA CGT TCA CC) e GH20 (GAA GAG CCA AGG ACA GGT AC), which amplified the segment of 268 base pairs (pb) of the human β-globin gene, ensuring the qualification and quantification of DNA for HPV analysis, and by the PGM09 and PGMY11 oligonucleotides, which amplify a segment of 450 pb of a preserved region of gene L1 of *Human Papillomavirus*[27]. The thermocycler, model PTC100 (MJResearch, Watertown, Mass.) was used for amplification; the parameters for denaturation, annealing and lengthening of the ribbons were the following: 95°C for 5 minutes, followed by 40 51°C cycles for 30 seconds, 55°C for 1 minute, 72°C for 1 minute and, finally, 72°C for 5 minutes. Negative and positive controls were included with all amplifications, and the negative control was constituted by all elements except genomic DNA; and the positive control was constituted by HPV DNA type 16, extracted from cells of the *SiHa *strain (Ludwig Institute for Cancer Research). Four μg of the molecular DNA of the DNA *φ X 174RF *HaeIII molecular weight marker were used. The presence or absence of HPV DNA fragments and β-globin amplified from the oligonucleotides was analyzed in 1.5% agarose gel, in buffer TBE 0.5× with 0.3% ethidium bromide (0.1 mg/μL solution), under ultraviolet light.

#### Viral typing

The HPV positive samples were submitted to a new type of PCR, specific for viral type identification. For this purpose the RFLP (Restriction Fragment Length Polymorphism) technique was used, according to the methodology described by Bernard et al (1994) [28]. The amplified product was digested by the BamHl, Ddel, Haelll, HinfI, PstI, RsaI and SauAIII enzymes and analyzed by vertical electrophoresis in 4% polyacrylamide gel (20.3% acrylamide, 0.7 bisacrylamide, 0.07% ammonium persulphate, TBE 1X TEMED 0.7 μL/mL – GibcoBRL). The pGEM (PROMEGA) was used as a molecular weight marker. Later the samples in polyacrylamide gel were stained with silver nitrate and the fragments obtained compared to the prototypes described by Bernard et al. (1994) [28].

#### Amplification by nested-PCR in region E6/E7 of the HPV

The nested multiplex PCR (NMPCR) assay combines degenerate E6/E7 consensus primers and type-specific primers (MY09/11 and GP5+/6+) for the detection and typing of HPV genotypes 6/11, 16, 18, 31, 33, 35, 39, 42, 43, 44, 45, 51, 52, 56, 58, 59, 66 and 68. With regard to sensitivity and performance with clinical samples, the novel NMPCR assay is a potentially useful tool for HPV DNA detection in epidemiologic and clinical follow-up studies, especially when accurate HPV typing and the detection of multiple HPV infections are required.

The samples were amplified during the first PCR reaction using the degenerated primers GP-E6-3F (GGG WGK KAC TGA AAT CGG T), GP-E6-5B (CTG AGC TGT CAR NTA ATT GCT CA) and GP-E6-6B (TCC TCT GAG TYG YCT AAT TGC TC), W being A/T; K, G/T; R, A/G; Y, C/T and N, A/C/G/T. These primers amplify a 630 pb region in the E6/E7 region of the 38 most common types of HPV. The nested-PCR reaction is specific and was performed for the following types: 6/11, 16, 18, 31, 33, 42, 52 and 58, which represent the most prevalent viral types in the region[29]. The primers used and the sizes of the amplified products are shown in table 1. The entire procedure, both the first reaction (PCR) and the second reaction (nested-PCR) occurred according to Sotlar et al., 2004[30].

**Table 1:** Sequences of type-specific nested PCR primers used in this study.

HPV genotype	Primer sequences	Amplicon (pb)
6/11	TGC AAG AAT GCA CTG ACC AC TGC ATG TTG TCC AGC AGT GT	334 pb*
16	CAC AGT TAT GCA CAG AGC TGC CAT ATA TTC ATG CAA TGT AGG TGT A	457 pb
18	CAC TTC ACT GCA AGA CAT AGA GTT GTG AAA TCG TCG TTT TTC A	332 pb
31	GAA ATT GCA TGA ACT AAG CTC G CAC ATA TAC CTT TGT TTG TCA A	263 pb
33	ACT ATA CAC AAC ATT GAA CTA GTT TTT ACA CGT CAC AGT GCA	398 pb
42	CCC AAA GTA GTG GTC CCA GTT A GAT CTT TCG TAG TGT CGC AGT G	277 pb
52	TAA GGC TGC AGT GTG TGC AG CTA ATA GTT ATT TCA CTT AAT GGT	229 pb
58	GTA AAG TGT GCT TAC GAT TGC GTT GTT ACA GGT TAC ACT TGT	274 pb

* Base pairs.

#### Transplacental transmission

In the study, the transplacental transmission of HPV was considered when HPV DNA type-specific agreement was observed between the samples: (1) mother (genital or peripheral blood), placental and newborn (buccal, body or cord blood); or (2) mother (genital or peripheral blood) and newborn (cord blood)[19].

#### Vertical HPV transmission

In the study, vertical HPV transmission was considered when HPV DNA was found in newborns (cord blood or nasopharyngeal aspirates or buccal and body).

### Statistical analysis

Statistical analyses were performed with the SPSS computer software package (version 12.0 for Windows). Frequency tables were analyzed by using the chi-square test, with Pearson and likelihood ratio tests for the significance of differences between the categorical variables. The 95% confidence interval (95% CI) was calculated where appropriate. Differences in the means of continuous variables between the groups were analyzed by using nonparametric tests. In all analyses probability values of < 0.05 were regarded as significant.

## Results

The study included 49 pairs of mothers and newborns.

### HPV DNA in maternal genitalia

HPV DNA was detected in 49 (77.8%) of the 63 pregnant women who underwent delivery. The most frequently detected types of HPV DNA were 6/11 (20.7%), 42 (15.9%), 16 (15.9%), 18 (11%), 58 (6.1%) and 31, 35 e 52 (3.7% each). Of these 54.9%, 1.2%, 40.2% and 3.7% were types considered to present a high carcinogenic risk, possible high risk, low risk and HPV DNA present but not classified for viral type respectively (Table 2). Genital infections produced by a single type of HPV DNA (38.8%), by two types of HPV DNA (30.6%) and more than two types of HPV DNA (30.6%) were identified.

**Table 2:** HPV types in maternal genital sample.

HPV DNA			
Type n = 18	Carcinogenic risk	Frequency n = 82	%

6/11	LR	17	20.7
42	LR	13	15.9
16	HR	13	15.9
18	HR	9	11
58	HR	5	6.1
31	HR	3	3.7
35	HR	3	3.7
52	HR	3	3.7
51	HR	2	2.4
54	LR	2	2.4
59	HR	2	2.4
26	PHR	1	1.2
33	HR	1	1.2
34	HR	1	1.2
45	HR	1	1.2
68	HR	1	1.2
70	LR	1	1.2
73	HR	1	1.2
NC*	-	3	3.7

The HPV types were identified by both multiplex PCR and nested multiplex PCR methods. *NC = HPV DNA positive but could not be classified by type. LR – Low-risk HPV genotypes (HPV type 6, 11, 40, 42, 43, 44, 54, 61, 70, 72, 81 and CP6108). HR – High-risk HPV genotypes (HPV type 16, 18, 31, 33, 35, 39, 45, 51, 52, 56, 58, 59, 68, 73 and 82). PHR – Probable high-risk HPV genotypes (HPV type 26, 53 and 66) [61].

### HPV DNA in the placenta

HPV DNA was detected in 12 placentas (24.5%) of the 49 HPV DNA positive pregnant women (HPV DNA+) who underwent delivery. The fetal side of the placenta presented HPV DNA+ in 5 cases (41.7%, n = 5/12), the maternal placental side in 2 cases (16.7%, n = 2/12), while in 5 cases (41.7%, n = 5/12) research for HPV DNA was positive on both sides of the placenta. The viral types identified in the placentas were 6/11 (50%, n = 6/12), 16 (25%, n = 3/12), 18 (16.7%, n = 2/12), 42, 52 and 58 (8.3%, n = 1/12 – each). The type specific HPV concordance among the genital/placental samples was 91.7% (n = 11/12). Seven placentas (58.3%, n = 7/12) presented viral types considered a high carcinogenic risk (types 16, 18, 52 and 58) and 2 placentas presented two different types of HPV DNA (Table 3).

**Table 3:** Clinical and laboratory history of genital HPV infection during pregnancy and delivery and distribution of HPV types in maternal, newborn and placental samples.

	Epidemiology maternal				HPV type in samples										
	Pregnancy		Delivery		Maternal		Placental						Newborn		
	
Case	HPV lesion type	HPV lesion site	Type	HPV lesion	Genital	Peripheral blood	Fetal side			Maternal side			Aspirates nasopharyngeal	Buccal and body	Cord blood
										
							Biopsy		Brush	Biopsy		Brush			
													
							Border	Central		Border	Central				

1	Warts	VV	V	No	6/11										
2	HGSIL	C	C	No	16+6/11				6/11	6/11		6/11		6/11	
3	HGSIL	C	C	Yes	16+31						16				
4	Warts	VV	V	No	16+42+54					16		42+16		42	
5	LGSIL	C	C	Yes	18										
6	Warts	VV	C	No	6/11+16+31										
7	Warts	C+VV+VG	V	No	6/11+42		6/11			6/11			6/11	6/11	
8	HGSIL	C	C	Yes	52		6/11					6/11+52	6/11		
9	LGSIL	C	V	Yes	42+51+NC*										
10	Warts	VV+VG	V	No	6/11										
11	LGSIL	C	V	No	18				18			18	18		18
12	Warts	VV	V+F	No	NC*										
13	LGSIL	C	C	Yes	6/11+42										
14	LGSIL	C	C	Yes	16+42+58				6/11				59		
15	Warts	C+VV+VG	V	Yes	6/11+42										
16	Warts	VV+VG	C	No	6/11										
17	HGSIL	C	C	Yes	18+51										
18	Warts	VV+P	C	No	42+59										
19	Warts	VV	V	No	6/11	6/11									
20	HGSIL	C	C	No	42+35										
21	Warts	VV	C	Yes	52										52
22	Warts	VV	C	Yes	34										
23	Warts	VV+VG	V	Yes	18										
24	Warts	VV	V	No	16+73	16									
25	Warts	VV+VG	C	No	68										
26	Warts	C	V	Yes	6/11+16			16							
27	Warts	C+VV+VG	V	No	16+58										
28	Warts	VV	C	Yes	6/11+33										
29	LGSIL	C	V	No	16										
30	LGSIL	C	V	Yes	52+42+58+54				58						
31	LGSIL	C	C	No	16										
32	Warts	VV	C	No	6/11										
33	HGSIL	C	V	Yes	18		18								
34	Warts	VV	V	Yes	11								11		
35	Warts	VV+VG	C	Yes	42									42	
36	LGSIL	C	V	Yes	16										
37	HGSIL	C	C	Yes	58									6/11+52	
38	HGSIL	C	V	No	6/11+18										
39	HGSIL	C	C	Yes	18+31										
40	Warts	VV	V	No	6/11				6/11						
41	Warts	VV	C	No	42+35+NC*										
42	LGSIL	C	C	Yes	42										
43	LGSIL	C	C	yes	16+18+42										
44	LGSIL	C+VV+VG	C	Yes	18+26										
45	Warts	VV	V	Yes	6/11+58+59		6/11		6/11	6/11				6/11	6/11
46	Warts	VV+VG	C	Yes	6										
47	Warts	VV	V	Yes	35										
48	Warts	VV	V	No	70										
49	Warts	VV+VG	V	No	6+45	58									

The HPV types were identified by both multiplex PCR and nested multiplex PCR methods. *NC = HPV DNA positive but could not be classified by type.

Type of delivery = C – cesarean section; V- vaginal; and V+ F – vaginal with forceps.

HPV lesion site = C – cervical; VG – vaginal; VV – vulva; P – perineal.

HPV lesion type = Warts – genital warts; LGSIL – low-grade squamous intraepithelial lesions; HGSIL – high-grade squamous intraepithelial lesions.

It was observed that seven (58.2%, n = 7/12) cases presented HPV DNA+ for the genital/placental/newborn samples and five (41.7%, n = 5/12) cases presented HPV DNA+ for the genital/placental samples with negative research for HPV DNA in newborns (NB).

### HPV DNA in newborns

HPV DNA was identified in eleven NB (22.4%, n = 11/49). Five NB had HPV DNA+ in samples of nasopharyngeal aspirate, six in buccal and body scrapings, and three in arterial cord blood (Table 3). The viral types identified were 6/11 (45.5%, n = 5/11), 42 (18.2%, n = 2/11), 52 (18.2%, n = 2/11), 18 and 59 (9.1%, n = 1/11 – each). Four NB (36.4%, n = 4/11) presented viral types considered a high carcinogenic risk (types 18, 52 and 59). In one NB two types of HPV DNA were detected (types 6/11 and 52).

Among the eleven cases of NB HPV DNA+, seven (63.6%, n = 7/11) presented HPV DNA+ for the genital/placental/NB samples. Six of these cases (85.7%, n = 6/7) were in concordance as to the type-specific HPV among the placental/NB samples and five cases (71.4%, n = 5/7) presented concordance as to the type specific HPV among the genital/placental/NB samples, suggesting the transplacental transmission of the virus (10.2%, n = 5/49).

No physical abnormalities or genital warts were observed in the 49 newborns.

Among the 11 cases of NB HPV DNA+ (vertical transmission), four (36.4%, n = 4/11) did not present transplacental infection due to virus (Table 3). Of these, one case presented type specific HPV concordance among the genital/arterial cord blood samples (HPV type 52) suggesting the possibility of transplacental transmission. Among the three other cases, two had type specific HPV concordance among the genital/NB samples (HPV types 11 and 42).

On the other hand, five NB (41.7%, n = 5/12) were negative for HPV DNA research, while in their respective placentas HPV DNA+ was shown (Table 3). The HPV identified were types 16 (40%, n = 2/5), 6/11, 18 and 58 (20%, n = 1/5 – each). Four NB (80%, n = 4/5) presented viral types considered a high carcinogenic risk (types 16, 18, 58). The concordance of type specific HPV observed among the genital/placental samples was 100% (n = 5/5).

### HPV DNA in arterial cord blood

Studying the arterial blood from the umbilical cords of NB (Table 3), 3 cases (6.1%, n = 3/49) HPV DNA+ for viral types 6/11, 18 and 52 were observed. In 2 clinical cases there was concordance of type specific HPV among the genital/placental/arterial cord blood samples, and in the other case, concordance of type specific HPV among the genital/arterial cord blood was observed. The latter case mentioned, which corresponds to the same case mentioned above, was considered transplacental transmission (hematogenic, directly through the placenta, without any infection in the latter). Of the 3 cases studied, two (66.7%) had HPV DNA types 18 and 52 considered a high carcinogenic risk.

### HPV DNA in maternal peripheral blood

Three (6.1%, n = 3/49) parturients had HPV DNA in their peripheral blood (Table 3). In two cases HPV DNA that was considered a high carcinogenic risk (types 16 and 58) was detected. There was 66.7% (n = 2/3) concordance of type specific HPV among the maternal genital/peripheral blood samples. In all three cases no HPV DNA was identified in the respective placentas and NB.

Statistical analysis showed a significant association between placental HPV infection and the epidemiological variable history of immunodepression (HIV, p = 0.011), as observed in table 4 and 5.

**Table 4:** HPV status of the placenta and maternal factors.

**Maternal variable**	**Placental HPV DNA infection**			
	
	**Positive (n = 12)**		**Negative (n = 37)**	
**Age (years)**				
≤ 19	6	(50%)	17	(45.9%)
≥ 20 to ≤ 29	3	(25%)	12	(32.4%)
≥ 30 to ≤ 39	2	(16.7%)	7	(18.9%)
≥ 40 to ≤ 49	1	(8.3%)	1	(2.7%)
Mean for placental HPV DNA positive group (24.3 ± 8.3 years)	-	-	-	-
Mean for placental HPV DNA negative group (23.8 ± 8.2 years)	-	-	-	-
				
**Race**				
White	11	(91.7%)	33	(89.2%)
Non-white	1	(8.3%)	4	(10.8%)
				
**Level of education**				
Illiterate	-	-	-	-
Elementary (complete or incomplete)	6	(50%)	22	(59.4%)
High school (complete or incomplete)	5	(41.7%)	15	(40.5%)
College (complete or incomplete)	1	(8.3%)	-	-
				
**Smoking**				
No	10	(83.3%)	24	(64.9%)
< 10 cigarettes per day	-	-	6	(16.2%)
≥ 10 cigarettes per day	2	(16.7%)	7	(18.9%)
				
**Marital status**				
Married	3	(25%)	8	(21.6%)
Single	2	(16.7%)	9	(24.3%)
Cohabiting	6	(50%)	19	(51.4%)
Divorced, separated	1	(8.3%)	1	(2.7%)
				
**Marital stability (years)**				
≤ 2	8	(66.7%)	26	(70.3%)
≥ 3 to ≤ 5	3	(25%)	7	(18.9%)
≥ 6	1	(8.3%)	4	(10.8%)
Mean for placental HPV DNA positive group (3.1 ± 4.3 years)	-	-	-	-
Mean for placental HPV DNA negative group (2.8 ± 4.7 years)	-	-	-	-
				
History of Immunodepression (HIV)*				
No	10	(83.3%)	37	(100%)
Yes	2	(16.7%)	-	-

Data are reported as number and percentage (in parentheses) of placental positive or negative infection for human papillomavirus. *P < 0.011 indicates a statistically significant difference between the positive and negative groups by Pearson's chi-square test (HIV – acquired immunodeficiency syndrome).

**Table 5:** HPV status of the placental and delivery factors.

**Maternal variable**	**Placental HPV DNA infection**			
	
	**Positive (n = 12)**		**Negative (n = 37)**	
**Type of HPV lesion**				
Genital warts	5	(41.7%)	23	(62.2%)
LGSIL^1^	3	(25%)	9	(24.3%)
HGSIL^2^	4	(33.3%)	5	(13.5%)
				
**Site of HPV lesion**				
Uterine cervix	8	(66.7%)	13	(35.1%)
Vulva	3	(25%)	13	(35.1%)
Vulva + vagina	-	-	7	(18.9%)
Vulva + perineal region	-	-	1	(2.7%)
Uterine cervix + vulva + vagina	1	(8.3%)	3	(8.1%)
				
**Type of HPV Infection**				
Single	3	(25%)	16	(43.2%)
Double	2	(16.7%)	13	(35.1%)
Multiple	7	(58.3%)	8	(21.6%)
				
**Type of delivery**				
Vaginal	8	(66.7%)	15	(40.5%)
Vaginal + forceps	-	-	1	(2.7%)
Cesarean section	4	(33.3%)	21	(56.8%)
Mean gestational age of the delivery in the placental HPV DNA positive group (39.7 ± 1.1 weeks)				
Mean gestational age of the delivery in the placental HPV DNA negative group (39.2 ± 2.4 weeks)				
				
**Gestational age at the time HPV infection was diagnosed (week)**				
≥ 4 to ≤ 12	2	(16.7%)	17	(45.9%)
≥ 13 to ≤ 28	4	(33.3%)	8	(21.6%)
≥ 29 to ≤ 42	1	(8.3%)	6	(18.9%)
Prior to pregnancy	5	(41.7%)	5	(13.5%)
Mean in the placental HPV DNA positive group (10.5 ± 13.3 weeks)				
Mean in the placental HPV DNA negative group (14.63 ± 12 weeks)				
				
**Time of RUPREME^3 ^(min)**				
≤ 360	11	(100%)	35	(92.1%)
≥ 361 to ≤ 720	-	-	1	(2.6%)
≥ 721	-	-	2	(5.3%)
Mean of placental HPV DNA positive group (37 ± 37 minutes)	-	-	-	-
Mean of placental HPV DNA negative group (106 ± 244 minutes)	-	-	-	-
				
**Duration of labor (min)**				
≤ 240	6	(54.5%)	22	(57.9%)
≥ 241 to ≤ 360	2	(18.2%)	10	(26.3%)
≥ 361	3	(27.3%)	6	(15.8%)
Mean of placental HPV DNA positive group (236 ± 196 minutes)	-	-	-	-
Mean of placental HPV DNA negative group (185 ± 203 minutes)	-	-	-	-
				
**HPV lesion at delivery**				
Yes	7	(58.3%)	19	(51.4%)
No	5	(41.7%)	18	(48.6%)

Data are reported as number and percentage (in parentheses) of infection placental positive or negative for human papillomavirus. *P < 0.05 indicates a statistically significant difference between the positive and negative groups by Pearson's chi-square test. ^1^Low-grade squamous intraepithelial lesions. ^2^High-grade squamous intraepithelial lesions. ^3^RUPREME = rupture of membrane amniotic.

In the group of pregnant women negative for genital HPV DNA (n = 14/63), it was observed that all samples, both of maternal peripheral blood and those of nasopharyngeal aspirate, buccal and bodily scrapings and arterial cord blood and those of placental biopsies and scrapings presented negative results for HPV DNA research.

### HPV detection and typing methods

Evaluating the HPV DNA detection and typing methods, it was observed that the multiplex PCR methodology identified HPV DNA in 41 pregnant women (83.7%, n = 41/49), in 31 pregnant women (75.6%, n = 31/41) only a single type of HPV DNA was identified, and two or more types of HPV in 10 pregnant women (24.4%, n = 10/41). The nested multiplex PCR method (although it was used to identify and type 9 types of HPV shown as the most prevalent in the city of Caxias do Sul) identified HPV DNA in 43 pregnant women (87.8%, n = 43/49), only a single type of HPV DNA in 28 pregnant women (83.7%, n = 28/43), and two or more types of HPV in 15 pregnant women (83.7%, n = 15/43). Together the multiplex PCR and nested multiplex PCR methods identified HPV DNA in 49 pregnant women (100%, n = 49/49), only a single type of HPV DNA in 19 pregnant women (38.8%, n = 19/49) and two or more types of HPV in 30 pregnant women (61.2%, n = 30/49).

The multiplex PCR method identified HPV DNA in only two newborns (18.2%, n = 2/11), while the nested multiplex PCR method identified it in 9 newborns (81.8%, n = 9/11).

In the placentas, multiplex PCR identified HPV DNA in only a single one (83.7%, n = 1/12), while the nested multiplex PCR method identified HPV in 12 cases (100%, n = 12/12).

## Discussion

Human papillomavirus infection is one of the most frequent sexually transmitted diseases [31-33]. Non-sexual transmission[34] of HPV may occur directly by contact with the skin or mucosas (between people or by self-inoculation), or indirectly through contaminated objects, or still during the perinatal period.

Perinatal transmission may occur: (1) directly, during the passage of the fetus through the birth canal and on coming into contact with infected maternal secretions[13,18]; in delivery by cesarean section by ascending infection from the vaginal canal, after a premature rupture of the amniotic membranes [35]; in managing the mother with the baby (changing nappies, bathing)[10]; (2) indirectly, during vaginal delivery from contaminated objects; and (3) intrauterine transmission at the time of fertilization from sperm carrying latent HPV[36]; ascending infection from secretions of the maternal genital tract; and transplacental[11,19].

### HPV DNA in pregnant women

HPV DNA was detected in 49 pregnant women (77.8%, n = 49/63). The percentage found was considered high compared to the existing literature. However, given the origin of the population studied, from outpatient clinics dealing with prenatal examinations and infectious diseases, these figures were already expected. The data regarding the prevalence of HPV infection in pregnancy are highly discordant: 5.4% reported by Tenti et al. (1997)[37] and 68.8% mentioned by Cason et al. (1995)[15]. The diversity of percentages observed is related to different factors that by themselves could influence the results, such as: diagnostic techniques, the characteristics of the samples and the inclusion criteria. Eppel et al. (2000)[16] observed a 24.6% prevalence of HPV infection in the uterine cervix of pregnant women. Recently, Takakuwa et al. (2006)[38], examining the cervical smears of 1.183 pregnant women for HPV DNA using the PCR-RFLP methods, observed a prevalence of 22.6% in pregnant women aged less than 25 years. This percentage was statistically significant (p < 0.0005) compared to the percentage obtained in pregnant women over the age of 25 years (11.3%), and it was concluded that the prevalence of HPV is considered high in young Japanese pregnant women.

Studying the type of lesion produced by HPV in the maternal genitalia, it was observed that 57.1% had genital warts, 24.5% low grade cervical intraepithelial lesions, and 18.4% high grade cervical intraepithelial lesions, results which could suggest a higher percentage of HPV DNA considered a low carcinogenic risk, which, however, was not observed. Of the HPV DNA types detected 54.9%, 1.2% and 40.2% were viral types considered a high carcinogenic risk, possible high risk and low risk, respectively. Genital infections produced by two or more types of HPV DNA were identified in 61.2% of the cases. Lu et al. (2003)[39] studying the prevalence and viral type in pregnant women with a diagnosis of squamous atypias of the uterine cervix detected HPV DNA in 88.6% of the cases. Of the HPV positive cases, 79.6%, 4.3% and 5.4% were considered a high carcinogenic risk, probable high risk and low risk, respectively. The most frequent viral types detected were 52 (31.2%), 16 (15.1%), 39 (11.8%), 53 (10.8%), and 18 and 58 (9.7% each). Viral infection by multiple types was detected in 43% of the cases. Hernandez-Giron et al. (2005)[40], in a population study in México detected high carcinogenic risk HPV DNA in 37.2% of 274 pregnant women and 14.2% of 1,060 non-pregnant women.

Infections by multiple types of HPV are considered relatively common among the population in general[41]. Thomas et al. (2000)[42] reported that infection by multiple types of HPV are acquired more frequently than expected. These authors suggested that populations with a specific sexual behavior of exposing themselves to an ensemble of different types of HPV, or else the preexistence of a type of HPV could make it easier to acquire a new type of virus through an as yet unknown mechanism. Other authors[43] disagreed with the above statements and suggested that the risk factors are the same, both to acquire a single infection or a multiple one for HPV. A few authors suggested several hypotheses to account for the high rates of HPV infection observed in pregnant women, such as the immunosuppressive and hormonal states induced by pregnancy[40,44]. These hypotheses could also explain the rate of multiple HPV infection (61.2%) observed in this study.

### HPV DNA in the placenta

The use of different methods to sample the placentas was determinant for a more accurate identification of HPV DNA in third trimester pregnancy placentas (24.5%, n = 12/49). The results show that the isolated use of scraping methods or biopsies, especially if applied only to one of the sides of the placental disk, would detect a smaller number than the total obtained in this study.

The viral types identified in the placentas were 6/11 (50%, n = 6/12), 16 (25%, n = 3/12), 18 (16.7%, n = 2/12), 42, 52 and 58 (8.3%, n = 1/12 – each). The HPV DNA identified in the placentas were 6/11, 16, 18, 52 and 58. Seven placentas (58.3%, n = 7/12) presented HPV considered a high carcinogenic risk (types 16, 18, 52 and 58) and two (16.7%, n = 2/12) presented two different types of HPV DNA. The presence of HPV DNA in the placenta indicates the possibility of transplacental exposure to viral infection and to the need of considering the possible consequences of this exposure during the period: (1) intrauterine, to miscarriages[45] and possible malformations[16]; (2) postnatal period to genital warts in childhood[46], in adolescence juvenile-onset recurrent papillomatosis[5]; (3) in lifetime, the possible transmission of the carcinogenic agent[47,48].

In addition, the concordance observed in the type specific HPV between the genital/placental samples (91.7%, n = 11/12), strongly suggests that the HPV DNA detected in the placenta comes from maternal viral infection. This placental infection could be the result of an ascending canalicular infection from genital secretions (transamniotic) or hematogenic. The difference found in the types of HPV DNA may be due to different causes, such as contamination of the samples (unlikely), infection from the semen at the time of fertilization, infection due to multiple types, or subtypes and/or variants of HPV. Eppel et al. (2000)[16] in their study on HPV DNA detection in placentas, did not identify them in any of the 147 samples of chorionic vilosity collected by transabdominal amniocentesis. Even so, the authors suggested the possibility of transplacental viral transmission.

### HPV DNA in newborns

As seen in the evaluation of methods to sample the placenta, the use of different sampling methods in the NB was determinant for a more precise identification of the percentage of vertical transmission of HPV (22.4%, n = 11/49). The results show that the isolated use of oral and bodily cavity scraping methods, or nasopharyngeal aspirates, or arterial cord blood, if applied individually for clinical screening would detect a smaller number of NB HPV DNA+ than the total obtained in this study.

The viral types identified in the NB were 6/11 (45.5%, n = 5/11), 42 (18.2%, n = 2/11), 52 (18.2%, n = 2/11), 18 and 59 (9.1%, n = 1/11 – each). Genital warts, which are caused by HPV types 6 or 11, are considered a frequent complication in pregnancy and clinically important due to the possibility of vertical transmission. Armstrong et al. (2000)[49] considered juvenile recurrent respiratory papillomatosis a consequence of vertical transmission of HPV. However, the risk of developing this complication in a child born to a mother infected with HPV is one to several hundred exposures[50]. Smith et al. (1995)[51] showed a rate of only 1% of vertical transmission of HPV DNA. Other authors reported higher percentages, using the PCR methodology for HPV type 16 and 18 in genital scrapings and oral cavity of mother/NB pairs, respectively, and detected vertical transmission rates between 31% and 73%[13,15,18,52,53].

Four NB (36.4%, n = 4/11) presented viral types considered a high carcinogenic risk (types 18, 52 and 59) and one presented two different types of HPV DNA (types 6/11 and 52). These data are sufficient evidence to confirm the perinatal transmission of HPV, considered a high carcinogenic risk. These findings require future studies to be able to establish: (1) the significance and consequences of infection in the child; (2) their relationship with the infections detected in adults; (3) the risk for the development of cancer in lifetime. The virus infects mainly the epithelial cells, where it may remain latent for a very long time, evolve to the subclinical form, and thus remain, or reactivate, with a resulting accumulation of chromosomal mutations in host cells. The next result after this accumulated latent carcinogenic potential of certain types of HPV during childhood would be the development of a neoplasm in lifetime. The natural history of papilloma infection is characterized by regression in a period that varies from months to years[54].

In 5 cases (41.7%, n = 5/12) concordance of type specific HPV was observed between the genital/'placental/NB samples and in 1 case (8.3%, n = 1/12) between the genital/arterial cord blood samples, suggesting that there is often placental transmission (50%, n = 6/12). This was the first study in third trimester placentas to suggest the percentage of transplacental transmission of HPV DNA.

Several authors have focused special attention on the mode of HPV transmission. In 1992, Tseng et al.[19] suggested transplacental transmission of the virus, after detecting the same viral genome (HPV type 16) in cervicovaginal smears, in mononuclear cells of peripheral blood of fifteen pregnant women and in the cord blood of seven newborns from these same mothers. Favre et al. (1998)[11] showed the presence of several types of HPV DNA in amniotic liquid, placental cells and cervicovaginal smears of a mother and newborn with *epidermodisplasia verruciforme*. Hermonat et al. (1997)[25] recorded that the infection of HPV was three times more prevalent in specimens of spontaneous abortion in the first trimester. Later, in 1998, the same authors[55], confirmed the presence of HPV DNA in placental tissue of spontaneous abortions and concluded that the predominant site for HPV DNA type 16 findings were the cells of the syncytiotrophoblast. Thus, they raised the hypotheses of viremia, not yet convincingly documented, and of contamination of placental cells by oocyte infection before or right after implantation, by ascending infection or by infection carried by sperm containing latent HPV.

This study pointed to five cases of NB (41.7%, n = 5/12) negative for the research of HPV DNA, while in their respective placentas HPV DNA was detected. Four of these placentas presented viral types considered a high carcinogenic risk (types 16, 18, 58), and 100% (n = 5/5) of type specific HPV concordance is observed among the genital/placental specimens. The results achieved show that transmission of the virus to the fetus is not a prerogative of every HPV DNA+ woman, or in all cases of HPV DNA+ placentas, pointing to the existence of other as yet unknown factors that could be involved in transplacental transmission. Sedlacek et al. (1989)[56] detected the presence of HPV DNA in the oral cavity of 36.5% of the newborn, delivered vaginally to mothers with a diagnosis of HPV DNA+ for cervical cells. Kaye et al. (1994)[57] showed that the pregnant women who transmitted the virus to their concepts had a higher viral load.

Among the eleven cases of RN HPV DNA+ (vertical transmission), four cases (36.4%, n = 4/11) did not present HPV DNA in their respective placentas. One case out of this total presented type-specific HPV concordance between the genital/arterial cord blood (HPV type 52) and two presented concordance of type specific HPV between the genital/NB samples (HPV types 11 and 42). These results emphasized the possibility that other HPV transmission routes exist during pregnancy (transamniotic ascending infection), or during labor (ascending infection after the amniotic membranes are ruptured), or during delivery (by the fetus passing through the contaminated birth canal).

### HPV DNA in arterial cord blood and HPV DNA in maternal peripheral blood

Three cases of NB (6.1%, n = 3/49) who presented HPV DNA+ in arterial cord blood samples were seen. The three cases were considered transplacental transmission due to finding concordance of the type specific HPV among the genital/NB samples. In these three cases, as in all cases of vertical transmission, no HPV DNA was detected in maternal peripheral blood. On the other hand, three cases (6.1%, n = 3/49) were also observed of parturients who had HPV DNA in their peripheral blood, without HPV DNA in the respective placentas and NB. These results suggest that HPV infections in the placentas may have occurred by another route, which was not hematogenic, either by an infection that was already present before pregnancy in the endometrium, or by an ascending infection during the egg implantation period, or at the time of fertilization by sperm contaminated by the virus, or else during pregnancy facilitated by the uterine anatomy. The HPV predilection for tissues, apparently exclusive to the pavement epithelium of the skin and the mucosas, has been challenged in the last few years, since several studies demonstrated the capacity of HPV to infect different sites. The studies of Teseng et al. (1992)[19] may be mentioned, who demonstrated the presence of HPV in the amniotic liquid of pregnant women before labor, and Hermonat et al. (1998)[55] who described the presence of HPV in the syncytiotrophoblast of spontaneous abortions, proving the capacity of HPV to locate also in the uterine cavity. These studies showed the capacity of the virus to infect the uterine cavity, and therefore it is no surprise that the virus appears in the endometrium. Fedrizzi et al. (2004)[58] found HPV DNA (types 16 and 18) in 10% of the women with a normal endometrium. The exception is the work by Lai et al. (1992)[59] who found HPV DNA in 70% of the cases studied that had a normal endometrium or some benign disease. However, O'Leary et al. (1998)[60] did not find HPV DNA in the normal endometrium.

The positive and significant correlation between placental HPV infection and the maternal epidemiological variable "history of immunodepression" (HIV, p = 0.011), may be related to the special characteristics of the gravid cycle, especially the changes in the hormonal and immunological balance prevailing during this period, which might favor placental HPV infection.

### HPV detection and typing methods

Although the nested multiplex PCR methodology is used to identify and type only 9 types of HPV shown as the most prevalent in the city of Caxias do Sul, it performed very well in identifying maternal HPV DNA, and also considerably increased the number of pregnant women with multiple virus infections. In the newborn and placental samples the nested multiplex PCR method showed its great sensitivity and specificity to identify HPV. The use of that method was also crucial to evaluate the concordance of type specific HPV DNA among the maternal/placental/newborn samples, thus defining the vertical and transplacental transmission rates of the virus.

Concluding, the HPV DNA detection rate in the placenta was 24.5% (n = 12/49) and the transplacental transmission rate was 12.2% (n = 6/49). A transplacental transmission rate of 54.5% (n = 6/11) was observed when only the cases of vertical transmission were analyzed. These results were achieved in analyses of the placentas and newborns of mothers with genital warts or intraepithelial lesions of the uterine cervix. Thus, different forms of management can be adopted for each of these different stages (pre-gestational, gestation, delivery and the first few months after delivery), both from the diagnostic and therapeutic perspective. The mother and the newborn must be observed clinically and educational preventive measures must be established concerning the forms of HPV transmission, besides effective strategies for specific immunization.

In future, the HPV DNA rates in must be observed in the normal endometrium of women of reproductive age, in order to explain the possible route of infection by continuity and/or contiguity between the endometrium and conception products.
